# Desformylflustrabromine, a positive allosteric modulator of α4β2-containing nicotinic acetylcholine receptors, enhances cognition in rats

**DOI:** 10.1007/s43440-020-00092-4

**Published:** 2020-03-23

**Authors:** Agnieszka Nikiforuk, Ewa Litwa, Martyna Krawczyk, Piotr Popik, Hugo Arias

**Affiliations:** 1 Department of Behavioral Neuroscience and Drug Development, Maj Institute of Pharmacology Polish Academy of Sciences, 12 Smetna Street, 31-343 Krakow, Poland; 2 Department of Pharmacology and Physiology, Oklahoma State University College of Osteopathic Medicine, Tahlequah, OK USA

**Keywords:** Α4β2-nAChRs, Cognition, Desformylflustrabromine, Positive allosteric modulators, Rat

## Abstract

**Rationale:**

The α4β2 nicotinic acetylcholine receptors (α4β2-nAChRs) may represent useful targets for cognitive improvement. It has been recently proposed that a strategy based on positive allosteric modulation of α4β2-nAChRs reveals several advantages over the direct agonist approach. Nevertheless, the procognitive effects of α4β2-nAChR positive allosteric modulators (PAMs) have not been extensively characterized.

**Objectives:**

The aim of the present study was to evaluate the procognitive efficacy of desformylflustrabromine (dFBr), a selective α4β2-nAChR PAM.

**Methods:**

Cognitive effects were investigated in the novel object recognition task (NORT) and the attentional set-shifting task (ASST) in rats.

**Results:**

The results demonstrate that dFBr attenuated the delay-induced impairment in NORT performance and facilitated cognitive flexibility in the ASST. The beneficial effects of dFBr were inhibited by dihydro-β-erythroidine, a relatively selective α4β2-nAChR antagonist, indicating the involvement of α4β2-nAChRs in cognitive processes. The tested α4β2-PAM was also effective against ketamine- and scopolamine-induced deficits of object recognition memory. Moreover, procognitive effects were also observed after combined treatment with inactive doses of dFBr and TC-2403, a selective α4β2-nAChR agonist.

**Conclusions:**

These findings indicate that dFBr presents procognitive activity, supporting the strategy based on α4β2-nAChR potentiation as a plausible therapy for cognitive impairment.

**Electronic supplementary material:**

The online version of this article (10.1007/s43440-020-00092-4) contains supplementary material, which is available to authorized users.

## Introduction

Converging lines of evidence indicate that nicotinic acetylcholine receptors (nAChRs) are involved in the regulation of cognitive processes as well as in the pathophysiology of disorders that affect cognitive abilities, such as schizophrenia and Alzheimer’s disease (AD) [1–3]. The two most predominant nAChRs in the brain are heteropentameric α4β2-nAChRs and homopentameric α7-nAChRs. Recently, studies on possible therapies for cognitive decline in schizophrenia and AD have focused primarily on α7-nAChRs (e.g., [3]). Nevertheless, experimental evidence also supports the involvement of α4β2-nAChRs in the pathogenesis of schizophrenia and AD [4–9]. For example, post-mortem studies showed that the density of α4β2-nAChRs was decreased in the hippocampus [4] and striatum [5] of schizophrenia patients. Schizophrenia patients also demonstrated lower cortical β2-nAChR availability associated with executive dysfunctions [6]. Post-mortem studies also indicated a loss of α4β2-nAChRs in AD [7]. Moreover, a reduction in α4β2-nAChRs in typical AD-affected brain regions, as revealed by positron emission tomography, occurs at an early stage of AD and might give prognostic information about a conversion from mild to severe cognitive impairment during the progression of AD [8]. Abnormalities in α4β2-nAChRs may be closely linked to histopathological hallmarks of AD, such as the accumulation of β-amyloid (Aβ) peptides in the brain. For example, in vivo results showed that the content of α4β2-nAChRs was decreased, whereas Aβ deposits were increased in the brains of AD patients compared to the brains of normal elderly subjects [9]. The active peptide Aβ1–42 may also directly affect nAChR function. More specifically, α4β2-nAChRs can be blocked by Aβ1–42, decreasing its neuronal functions [10]. In line with clinical data, the cognitive performance of mice lacking the β2 subunit was deteriorated [11, 12].

Considering these results, it is plausible that a decreased function or content of α4β2-nAChRs might produce cognitive deficits; consequently, an enhancement of its function should improve cognition. However, while selective α4β2-nAChR agonists enhanced cognition in a variety of animal models (review in [13]), these preclinical efforts have not been translated into clinically effective treatments. The clinical lack of efficacy of orthosteric nicotinic agonists might be due to potential cross activity, overdosing, receptor desensitization and/or upregulation [2, 14]. Thus, drug development has shifted towards the positive allosteric modulation of α4β2-nAChRs, proposed as an advantageous therapeutic strategy compared to the direct agonist approach [2, 15, 16]. Since positive allosteric modulators (PAMs) increase the response of the endogenous neurotransmitter acetylcholine (ACh), without activating the receptor per se, the temporal integrity of neurotransmission is preserved, and the risk of overdosing is limited. In this regard, α4β2-selective PAMs might produce beneficial activities without generating side effects, such as those related to receptor desensitization or receptor upregulation that may occur after the chronic administration of orthosteric agonists.

Although several α4β2-PAMs have been characterized, there is only one study thus far that has assessed the potential procognitive efficacy of NS9283 [17], the compound that was further characterized as an unorthodox α4/α4 site-selective agonist [16]. Another α4β2-selective PAM, desformylflustrabromine (dFBr) [18], has been previously shown to suppress nicotine self-administration in rats [19], ameliorates symptoms of nicotine withdrawal in mice [20] and attenuates compulsive-like behaviours in a mouse model of obsessive–compulsive disorder [21]. Nevertheless, to our knowledge, there is no study demonstrating the procognitive efficacy of this compound.

Therefore, the first objective of our study was to investigate whether dFBr increases cognition in rats by using two animal tests, i.e., the attentional set-shifting task (ASST) and the novel object recognition task (NORT), which determine cognitive flexibility and recognition memory, respectively. The second goal of this work was to determine whether α4β2-nAChRs are involved in the procognitive effects elicited by dFBr. In this regard, the activity of dFBr was challenged against dihydro-β-erythroidine (DHβE), a potent competitive antagonist of α4β2-nAChRs with higher selectivity for this receptor subtype than for α7 and α3β4-nAChRs [22]. Moreover, the ability of dFBr to ameliorate the object recognition deficits elicited by the N-methyl-D-aspartate receptor (NMDAR) antagonist ketamine or by the muscarinic receptor antagonist scopolamine was also assessed. Finally, the efficacy of combined administration of the tested PAM with an α4β2-selective agonist, TC-2403 [(E)-*N*-methyl-4-(3-pyridinyl)-3-butene-1-amine; also called RJR-2403] [23], was tested in the NORT.

## Materials and methods

### Animals

Male Sprague–Dawley rats (Charles River, Sulzfeld, Germany) weighing 280–350 g on arrival were housed in a temperature-controlled (21 ± 1 °C) and humidity-controlled (40–50%) colony room with a 12/12 h light/dark cycle (lights on at 06:00 h). The rats were group-housed (4–5 rats/cage). For the ASST, rats were subjected to a mild food restriction (17 g/day food pellets) for at least one week prior to the testing day. Behavioural testing was performed during the light phase of the light/dark cycle. The experiments were conducted in accordance with the European Guidelines for animal welfare (2010/63/EU) and were approved by the II Local Ethics Committee for Animal Experiments at the Institute of Pharmacology, Polish Academy of Science, Krakow, Poland.

### Attentional set-shifting task (ASST)

The ASST assesses cognitive flexibility, i.e., the ability to modify behaviour in response to the altering relevance of stimuli. In this paradigm, rats must select a bowl containing a food reward based on the ability to discriminate the odours or the media covering the bait [24]. The ASST requires rats to initially learn a rule and form an attentional “set” within the same stimulus dimensions. At the extra-dimensional (ED) shift stage, animals must switch their attention to a previously irrelevant stimulus dimension and, for example, discriminate between the odours and not between the media covering the bait. The animal’s performance at the ED stage is considered an index of cognitive flexibility.

*Apparatus.* Testing was conducted in a dimly illuminated (20 lx) Plexiglas apparatus (length x width x height: 38 × 38 × 17 cm) with the grid floor and wall dividing half of the length of the cage into two sections. During testing, one ceramic digging pot (internal diameter of 10.5 cm and a depth of 4 cm) was placed in each section. Each pot was defined by a pair of cues along with two stimulus dimensions. To mark each pot with a distinct odour, 5 μl of a flavouring essence (Dr. Oetker®, Poland or The Body Shop, UK) was applied to a piece of blotting paper fixed to the external rim of the pot immediately prior to use. A different pot was used for each combination of digging medium and odour; only one odour was ever applied to a given pot. The bait (one-half of a Honey Nut Cheerio, Nestle®) was placed at the bottom of the “positive” pot and buried in the digging medium. A small amount of powdered Cheerio was added to the digging media to prevent the rat from trying to detect the buried reward by its smell.

*Procedure.* As described previously (e.g., [25]), the procedure lasted 3 days for each rat.

Day 1, habituation: rats were habituated to the testing area and trained to dig in the pots filled with sawdust to retrieve the food reward. The rats were transported from the housing facility to the testing room where they were presented with one unscented pot (filled with several pieces of Cheerios) in their home cages. After the rats had eaten the Cheerio from the home cage pot, they were placed in the apparatus and given three trials to retrieve the reward from both of the sawdust-filled baited pots. With each exposure, the bait was covered with an increasing amount of sawdust. Animals that did not dig for a food reward over 3 consecutive daily sessions were excluded from the experiment.

Day 2, training: rats were trained on a series of simple discriminations (SDs) to a criterion of six consecutive correct trials. For these trials, the rats had to learn to associate the food reward with an odour cue (e.g., arrack vs. orange, both pots filled with sawdust) and/or a digging medium (e.g., plastic balls vs. pebbles, no odour). All rats were trained using the same pairs of stimuli. The positive and negative cues for each rat were presented pseudorandomly and equally. These training stimuli were not used again in later testing trials.

Day 3, testing: rats performed a series of discriminations in a single test session. The first four trials at the beginning of each discrimination phase were discovery trials, during which the animals were allowed to dig in both bowls. The first trial of the discovery period was not included in the six criterion trials. In the subsequent trials, each incorrect choice was recorded as an error. Digging was defined as any distinct displacement of the digging media with either the paw or the nose; the rat could investigate a digging pot by sniffing or touching without displacing material. Testing was continued at each phase until the rat reached the criterion of six consecutive correct trials, after which testing proceeded to the next phase.

In the simple discrimination involving only one stimulus dimension, the pots differed along one of two dimensions (e.g., digging medium). For the compound discrimination (CD), the second (irrelevant) dimension (i.e., odour) was introduced, but the correct and incorrect exemplars of the relevant dimension remained constant. For the reversal of this discrimination (Rev 1), the exemplars and the relevant dimension were unchanged, but the previously correct exemplar was now incorrect, and vice versa. The intra-dimensional (ID) shift was then presented, comprising new exemplars of both the relevant and irrelevant dimensions, with the relevant dimension remaining the same as previously described. The ID discrimination was then reversed (Rev 2) so that the formerly positive exemplar became the negative one. For the extra-dimensional (ED) shift, a new pair of exemplars was again introduced; however, this time, the relevant dimension was also changed. Finally, the last phase was the reversal (Rev 3) of the ED discrimination.

The following pairs of exemplars were used: Pair 1: odour: spicy vs. vanilla, medium: cotton wool vs. crumpled tissue; Pair 2: odour: lemon vs. almond, medium: shredded pipette tips vs. wooden sticks; and Pair 3: odour: rum vs. cream, medium: shredded papers vs. silk. The exemplars were always presented in pairs, and they varied so that only one animal within each treatment group received the same combination. The assignment of each exemplar in a pair as being positive or negative at a given phase and the left–right positioning of the pots in the test apparatus on each trial were randomized.

### Novel object recognition task (NORT)

The NORT in rodents [26] has been increasingly used as an ethologically relevant paradigm for the study of visual recognition memory. This test is based on the spontaneous exploration of novel and familiar objects. The test consists of two trials separated by an intertrial interval (ITI). During the first trial, two identical objects are presented. In the second trial, one of the objects is replaced with a novel object. Successful object recognition is indicated when an animal spends more time interacting with the novel object than with the familiar one in the retention trial. An ITI of 24 h was chosen as a model of natural forgetting based on our previously published studies [27], which demonstrated that, at this delay, Sprague–Dawley rats do not discriminate novel objects from familiar ones. Under conditions of ketamine- or scopolamine-induced deficits, an ITI of 1 h, at which the animals demonstrate intact object recognition, was used [28].

*Apparatus* The rats were tested in a dimly lit (25 lx) open field made of dull grey plastic (length × width × height: 66 × 56 × 30 cm). After each measurement, the floor was cleaned and dried.

*Procedure* The rats were habituated to the arena (without any objects) for 5 min 24 h prior to testing. The test comprised two 3-min trials separated by an inter-trial interval (ITI) of 24 h (or 1 h in the ketamine and scopolamine experiments). During the first trial (familiarization, T1), two identical objects (A1 and A2) were presented in opposite corners, approximately 10 cm from the walls of the open field. In the second trial (retention, T2), one of the objects was replaced with a novel object (A = familiar and B = novel). The animals were returned to the home cage after T1. The objects used included a glass bulb filled with gravel and a plastic bottle filled with sand. The heights of the objects were comparable (~ 12 cm), and both objects were heavy enough to not be displaced by the animals. Half of the animals from each group received the glass bulb as a novel object, and the other half received the plastic bottle. The location of the novel object (the left end versus the right end of the open field) in the recognition trial was counterbalanced across the experimental groups. The exploration of an object was defined by looking, licking, sniffing or touching the object while sniffing but not leaning against, standing or sitting on the object. Any rat spending less than 5 s exploring the two objects within 3 min of T1 or T2 was eliminated from the study. The behaviour of the rats was recorded using a camera placed above the arena and connected to the Any-maze® tracking system (Stoelting Co., Illinois, USA). An experimenter blinded to the treatment conditions manually assessed the exploration time. Additionally, the distance travelled was automatically measured using the Any-maze® tracking system. Based on the exploration time (E) of the two objects, a discrimination index was calculated as DI = (EB–EA)/(EA + EB).

### Drug administration

Desformylflustrabromine hydrochloride (dFBr, an α4β2-nAChR PAM; Tocris, Bristol, UK), dihydro-β-erythroidine hydrobromide (DHβE, an α4β2-nAChR antagonist; Tocris, Bristol, UK), (E)-N-methyl-4-(3-pyridinyl)-3-butene-1-amine (TC-2403, also called RJR-2403, an α4β2-nAChR partial agonist; Abcam Biochemicals, Cambridge, UK), and scopolamine (Sigma–Aldrich, Poznan, Poland) were dissolved in distilled water. Ketamine [aqueous solution (115.34 mg/mL), Vetoquinol Biowet, Gorzów Wielkoposki, Poland] was diluted in distilled water to the appropriate dosage. In general, the compounds were administered intraperitoneally (IP), except TC-2403, which was given subcutaneously (SC). The drugs or vehicle (saline) was administered at a volume of 1 ml/kg of body weight.

*ASST*: dFBr (0.1, 0.3, and 1.0 mg/kg) or vehicle was administered 30 min prior to the SD phase of the task. To determine the ability of 3.0 mg/kg DHβE to block the procognitive effects of 1.0 mg/kg dFBr, the compounds were administered simultaneously 30 min before testing. The total number of animals subjected to the ASST experiments was *N* = 50 (2 rats were excluded during training). The number of animals in each experimental group was *N* = 6. Each rat was tested only once.

*NORT:* dFBr (1.0 and 3.0 mg/kg) or vehicle was administered 30 min prior to the acquisition trial (T1). To determine the ability of 3.0 mg/kg DHβE to block the procognitive effects of 3.0 mg/kg dFBr, the compounds were administered simultaneously 30 min before T1.

In the experiments in which amnestic agents were used, dFBr (1.0 and 3.0 mg/kg) was first administered, followed by ketamine (20 mg/kg) or scopolamine (1.25 mg/kg) 30 min later; after an additional 45 min (ketamine) or 30 min (scopolamine), the acquisition trial (T1) was performed. In the drug interaction studies, an inactive dose of dFBr (1.0 mg/kg) in combination with an inactive dose of TC-2403 (0.01 mg/kg) was administered 30 min prior to T1. The total number of animals used in the NORT was N = 96. Because of low (< 5 s) object exploration, 3 rats were excluded from the analysis. Each rat was tested no more than twice, with a 7-day washout period between each of the two tests. No animal received the same treatment twice.

dFBr and TC-2403 doses were based on our preliminary experiments (see Supplement 1) and previous studies demonstrating drug-evoked procognitive or behavioural effects [19, 20, 25, 29]. Because the applied dose range was adjusted to demonstrate the minimal effective dose of the tested compounds, the dosage schedule differed between the ASST and NORT. DHβE was administered at a dose that has been previously demonstrated to block the procognitive effects of TC-2403 [25, 27]. The doses of ketamine and scopolamine, adopted from our published protocols [28, 30], have been demonstrated to produce reliable impairment using the NORT.

### Statistics

*ASST*. The number of trials required to achieve the criterion of six consecutive correct responses (i.e., trials to criterion, TTC) was recorded for each rat and for each discrimination phase of the ASST. Data were analysed using a mixed design ANOVA with dFBr treatment as a between-subject factor and discrimination phase (SD, CD, Rev 1, etc.) as a repeated measure. In the interaction studies, DHβE treatment was a second between-subject factor in the analysis.

*NORT*. The data on exploratory preference were analysed using mixed-design ANOVAs with treatment as a between-subject factor and object as a repeated measure. The DI data were analysed using one-way ANOVAs, and the distance travelled was analysed using mixed-design ANOVAs, with treatment as a between-subject factor and trials as a repeated measure. In the interaction studies, DHβE treatment was a second between-subject factor in the analysis.

Post hoc comparisons were performed using Newman–Keuls tests. The statistical analyses were performed using Statistica 12.0 for Windows. Statistical significance was set at *p* < 0.05.

## Results

### Desformylflustrabromine (dFBr) enhances rat cognition in an α4β2-dependent manner.

#### Attentional set-shifting task

**Fig. 1:**
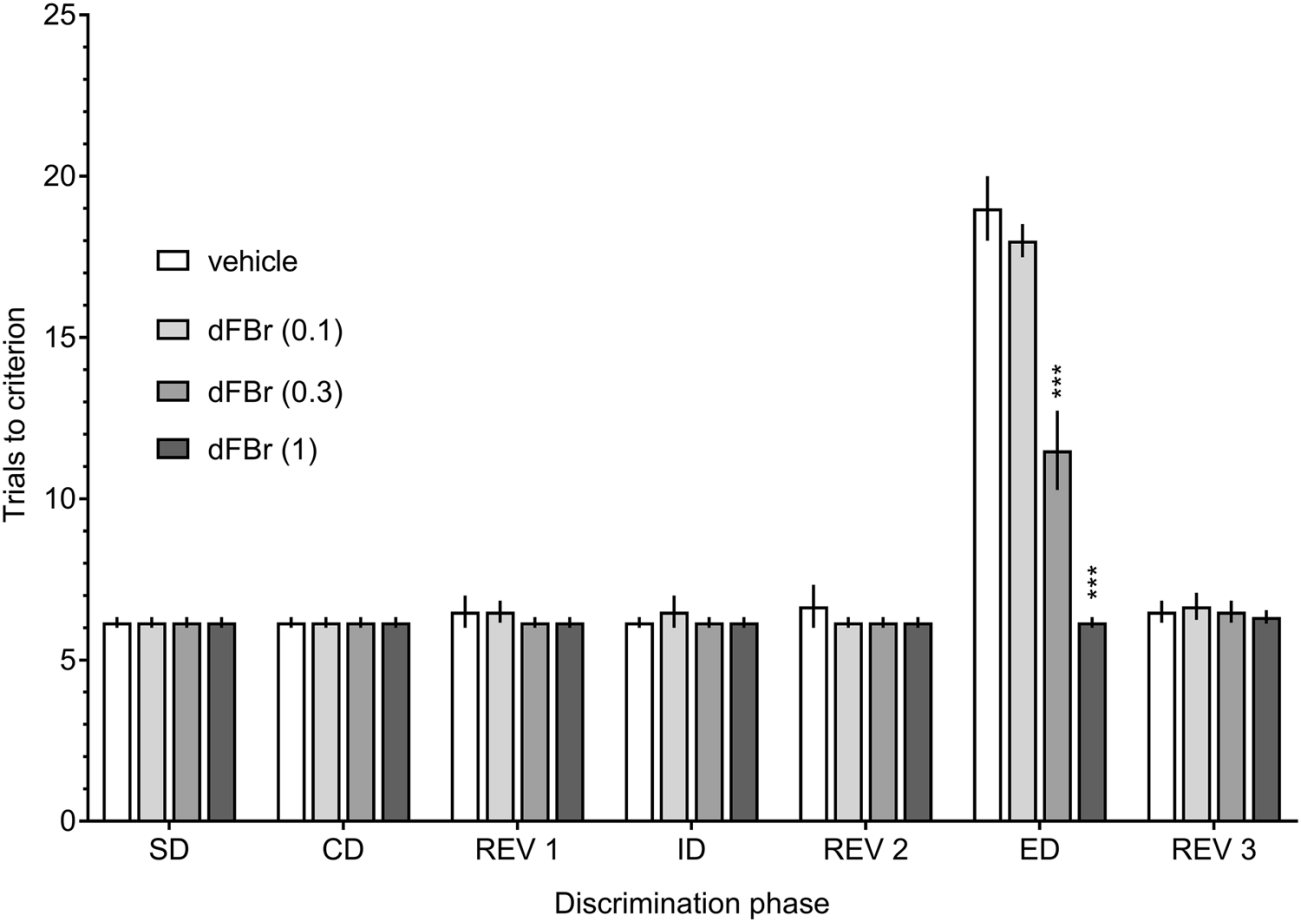
Dose–response effects of desformylflustrabromine on the attentional set-shifting task. Different doses of dFBr (0.1, 0.3, or 1.0 mg/kg) or vehicle were administered (IP) to rats 30 min prior to the test. Data are shown as the mean ± S.E.M. of the number of trials required to reach the criterion of six consecutive correct trials for each of the discrimination phases. *N* = 6 rats per group. ***p < 0.001, significant improvement in ED performance compared to that of the vehicle-treated group

The administration of dFBr, an α4β2-nAChR PAM, at doses of 0.3 and 1.0 mg/kg, but not at a dose of 0.1 mg/kg, reduced the number of trials to criterion in the ED phase compared to that in the vehicle-treated group (Fig. 1, a two-way ANOVA interaction: *F*[18,120] = 30.18; *p* < 0.001). There was no significant dFBr effect during any other test phase.

**Fig. 2:**
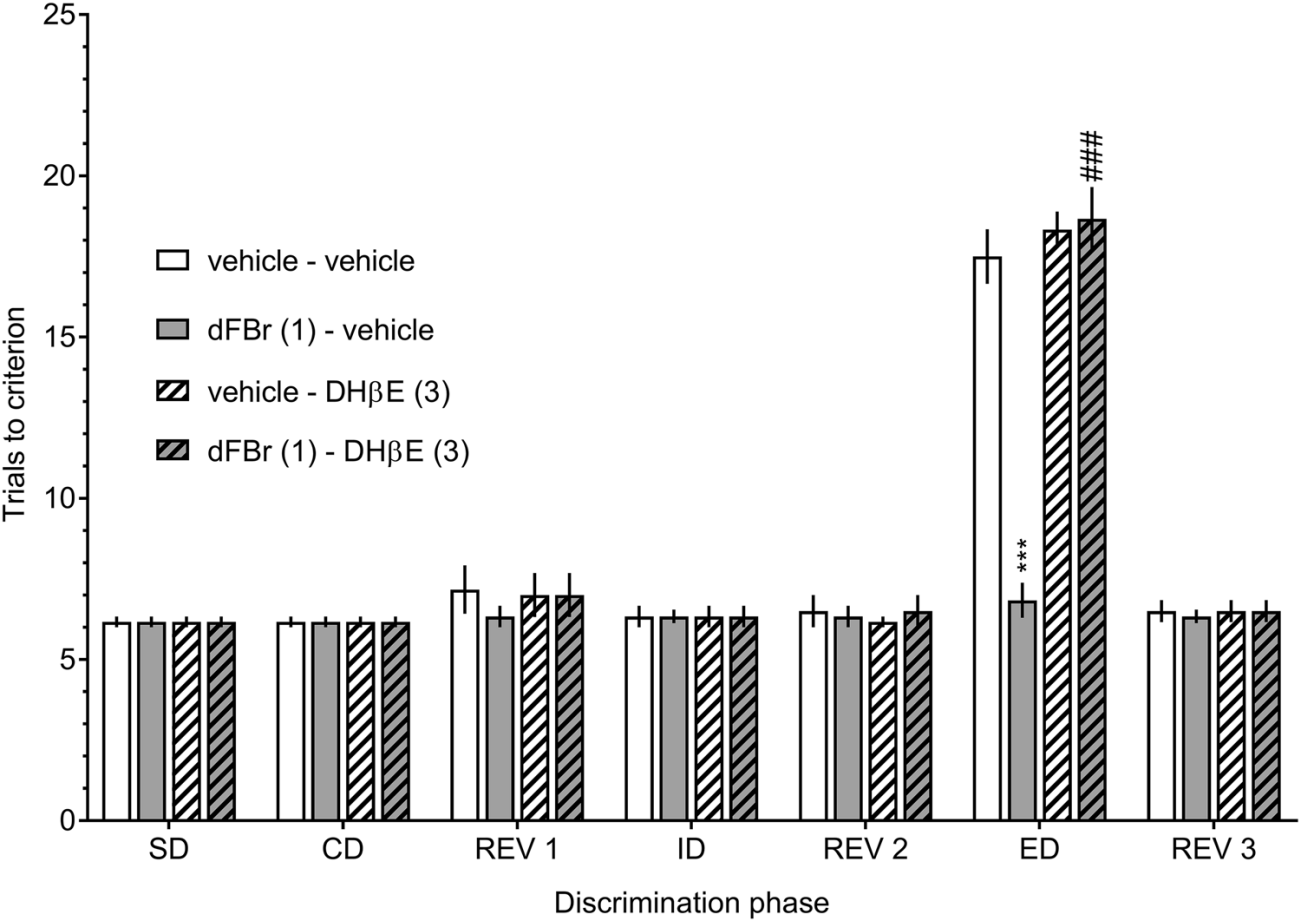
Dihydro-β-erythroidine inhibits the procognitive effects of desformylflustrabromine on the attentional set-shifting task. DHβE (3.0 mg/kg), dFBr (1.0 mg/kg), and their combinations were administered (IP) to rats 30 min prior to the test. Data are shown as the mean ± S.E.M. of the number of trials required to reach the criterion of six consecutive correct trials for each of the discrimination phases. *N* = 6 rats per group. ***p < 0.001, significant improvement in ED performance compared to the vehicle-treated group. ^###^*p* < 0.001, significant reduction in ED performance compared to the dFBr (1)-vehicle treated group

The cognitive enhancement elicited by 1.0 mg/kg dFBr was blocked by 3.0 mg/kg DHβE (an α4β2-nAChR antagonist), demonstrating that the observed effect was α4β2-dependent (Fig. 2, a three-way ANOVA interaction: *F*[6, 120] = 21.27, *p* < 0.001). The administration of 3.0 mg/kg DHβE alone did not affect rats’ ASST performance compared to the vehicle-treated group.

#### Novel object recognition task

No significant differences in the time spent exploring two identical objects in the acquisition phase in any group were observed (Supplementary Table 1 S2, a two-way ANOVA interaction for experiment 1 and a three-way ANOVA for experiment 2 were *F*[2, 25] = 0.39, NS and *F*[1, 28] = 2.22, NS, respectively).

**Fig. 3:**
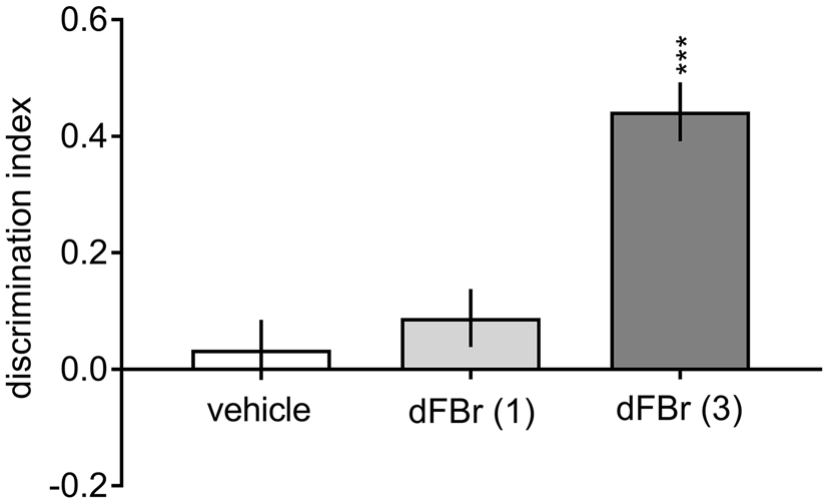
Dose–response effects of desformylflustrabromine on the novel object recognition task. Different doses of dFBr (1.0 or 3.0 mg/kg) or vehicle were administered (IP) to rats 30 min prior to the acquisition trial (T1). Data are shown as the mean ± S.E.M. of the discrimination index (DI) during the retention trial (T2) conducted 24 h after T1. *N* = 9–10 rats per group; ***p < 0.001 significant increase in DI compared to the vehicle-treated group

**Fig. 4:**
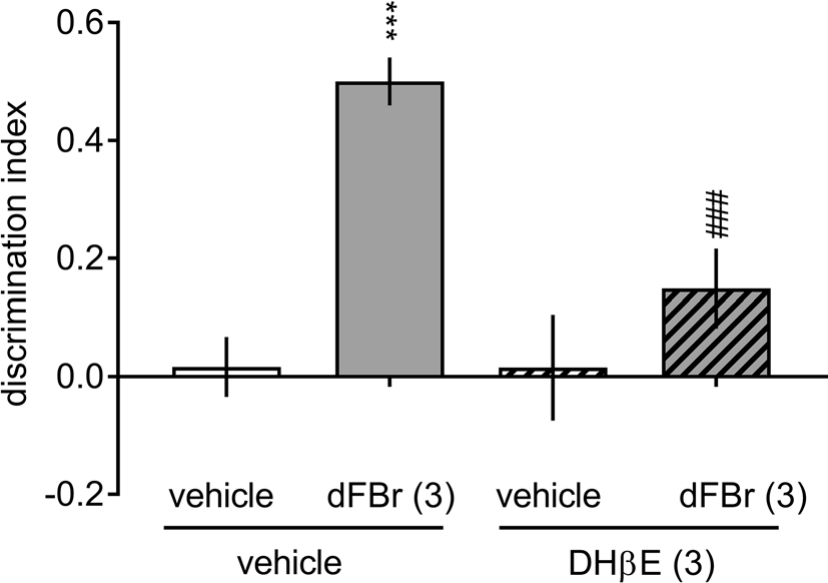
Dihydro-β-erythroidine inhibits desformylflustrabromine-increased recognition memory in the novel object recognition task. DHβE (3.0 mg/kg), dFBr (3.0 mg/kg), and their combinations were administered (IP) to rats 30 min prior to the acquisition trial (T1). Data are shown as the mean ± S.E.M. of the discrimination index (DI) during the retention trial (T2) conducted 24 h after T1. *N* = 8 rats per group. ***p < 0.001 significant increase in DI compared to that of the vehicle + vehicle-treated group; ^###^*p* < 0.001 significant reduction in DI compared to the vehicle + dFBr(3)-treated group

Vehicle-treated rats did not discriminate the novel object from the familiar object in the retention trial (Supplementary Table 1 S2; Fig. 3). This time-induced natural forgetting was ameliorated by the administration of 3.0 mg/kg dFBr (Supplementary Table 1 S2, two-way ANOVA interaction: *F*[2, 25] = 30.99, *p* < 0.001). Moreover, the DI for dFBr (3.0 mg/kg)-treated rats was significantly higher than that for vehicle-treated rats (Fig. 3, a one-way ANOVA: *F*[2, 25] = 19.68, *p* < 0.001). Similar procognitive efficacy was demonstrated for an α4β2-nAChR agonist, TC-2403, at doses of 0.1 and 0.3 mg/kg (detailed description is provided in Supplement 1). Interestingly, DHβE (3.0 mg/kg) blocked the procognitive effect elicited by dFBr {a three-way ANOVA interaction for exploration time: *F*[1, 28] = 15,47, *p *< 0.01 (Supplementary Table 1 S2) and a two-way ANOVA interaction for DI: *F*[1, 28] = 7.25, *p * < 0.05 (Fig. 4)}.

No significant treatment effects were observed for the distance travelled by the rats in the familiarization and retention trials (a two-way ANOVA interaction for experiment 1 and a three-way ANOVA for experiment 2 were *F*[2, 25] = 0.82, NS and *F*[1,28] = 0.85, NS, respectively, Supplementary Table 2 S2).

### Desformylflustrabromine reverses ketamine- and scopolamine-induced novel object recognition deficits

**Fig. 5:**
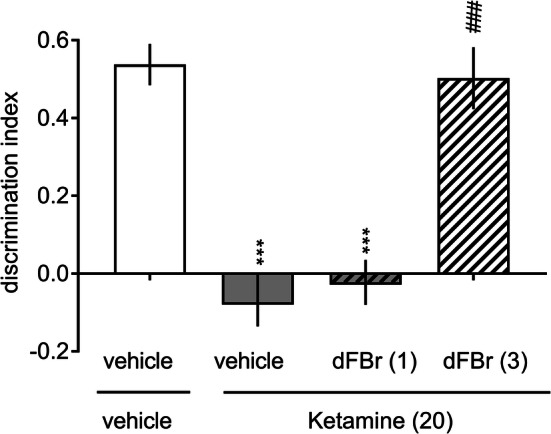
Desformylflustrabromine reverses ketamine-induced recognition memory deficits. Ketamine (20 mg/kg) was administered (IP) 45 min prior to the acquisition trial (T1), and dFBr (1.0 or 3.0 mg/kg) was administered (IP) 30 min prior to the ketamine injection. Data are shown as the mean ± S.E.M. of the discrimination index (DI) during the retention trial (T2) conducted 1 h after T1. *N* = 8 rats per group. ***p < 0.001 significant reduction in DI compared to that of the vehicle + vehicle-treated group; ^###^*p* < 0.001 significant increase in DI compared to that of the vehicle + ketamine-treated group

**Fig. 6:**
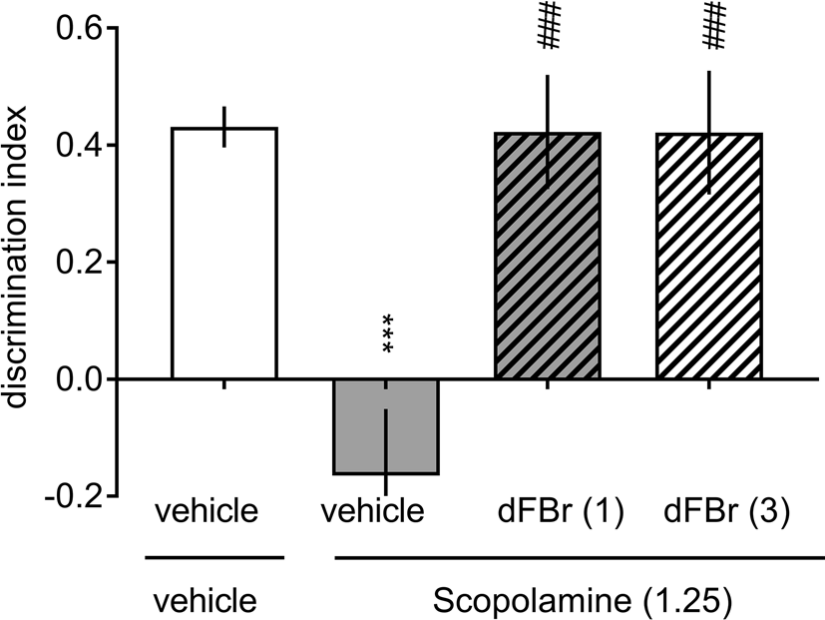
Desformylflustrabromine reverses scopolamine-induced recognition memory deficits. Scopolamine (1.25 mg/kg) was administered (IP) 30 min prior to the acquisition trial (T1), and dFBr (1.0 or 3.0 mg/kg) was administered (IP) 30 min prior to the scopolamine injection. Data are shown as the mean ± S.E.M. of the discrimination index (DI) during the retention trial (T2) conducted 1 h after T1. *N* = 8–10 rats per group. ***p < 0.001 significant reduction in DI compared to that of the vehicle + vehicle-treated group; ^###^*p* < 0.001 significant increase in DI compared to that of the vehicle + scopolamine-treated group

There were no significant differences in the time spent exploring two identical objects in the acquisition phase in any experimental group (Supplementary Table 1 S2, a two-way ANOVA interactions for ketamine and scopolamine studies were *F*[3, 28] = 1.82, NS and *F*[3, 29] = 0.71, NS, respectively). However, the administration of either 20 mg/kg ketamine (Fig. 5) or 1.25 mg/kg scopolamine (Fig. 6) abolished the ability of the animal to discriminate novel and familiar objects in the retention trial. Interestingly, the ketamine-induced deficit was reversed after treatment with 3.0 mg/kg dFBr (a two-way ANOVA interaction for exploration time: *F*[3, 28] = 22.28, *p* < 0.001; Supplementary Table 1 S2 and a one-way ANOVA interaction for DI: *F*[3, 28] = 26.47, *p* < 0.001; Fig. 5). Moreover, dFBr (1.0 or 3.0 mg/kg) reversed the impairing effects elicited by scopolamine (a two-way ANOVA interaction for exploration time: *F*[3, 29] = 8.99, *p* < 0.001; Supplementary Table 1 S2, and a one-way ANOVA interaction for DI: *F*[3, 29] = 10.76, *p* < 0.001; Fig. 6).

No significant treatment effects were observed on the distance travelled by rats in the familiarization and retention trials (a two-way ANOVA interactions for ketamine and scopolamine were *F*[3, 28] = 0.83, NS and *F*[3, 28] = 0.29, NS, respectively; Supplementary Table 2 S2).

### The co-administration of inactive doses of dFBr and TC-2403 facilitates novel object recognition memory

**Fig. 7:**
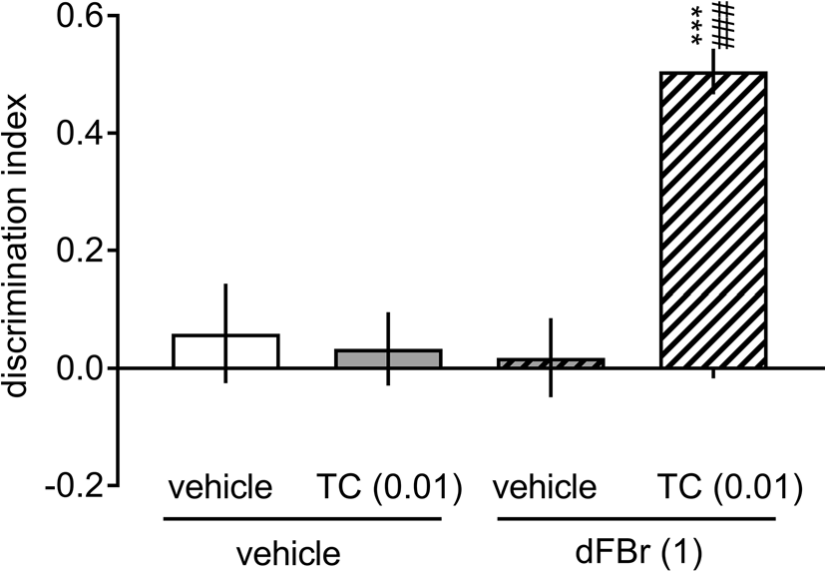
Effects of co-administration of inactive doses of TC-2403 and desformylflustrabromine on recognition memory. dFBr (1.0 mg/kg, IP), TC-2403 (0.01 mg/kg, SC), and their combination were administered to rats 30 min prior to the acquisition trial (T1). Data are shown as the mean ± S.E.M. of the discrimination index (DI) during the retention trial (T2) conducted 24 h after T1. *N* = 10–12 rats per group. ***p < 0.001 significant increase in DI compared to that of the vehicle + vehicle-treated group; ^###^*p* < 0.001 significant increase in DI compared to the drug alone-treated groups (i.e., vehicle + TC-2403 and vehicle + dFBr)

There were no significant differences in the time spent exploring two identical objects in the acquisition phase in any experimental group (Supplementary Table 1 S2, a two-way ANOVA interaction: *F*[1, 38] = 0.01, NS). The co-administration of an inactive dose of TC-2403 (0.01 mg/kg) with an inactive dose of dFBr (1.0 mg/kg) facilitated object recognition in the retention trial {a three-way ANOVA interaction for exploration time: *F*[1, 28] = 19.26, * p *< 0.001 (Supplementary Table 1 S2), and a two-way ANOVA interaction for DI: *F*[1, 38] = 16.31, *p* < 0.001 (Fig. 7)}. No significant treatment effects were observed for the distance travelled by rats in the familiarization and retention trials (a two-way ANOVA interaction: *F*[3, 38] = 1.58, NS, Supplementary Table 2 S2).

## Discussion

The present study demonstrated for the first time that dFBr facilitates cognitive flexibility and recognition memory in rats. Interestingly, the procognitive activities elicited by dFBr on the ASST and NORT were blocked by DHβE, a potent and relatively selective α4β2-nAChR antagonist, demonstrating that the main targets for the action of the tested PAM are α4β2-dependent. Moreover, the tested α4β2-PAM also reversed ketamine- and scopolamine-induced deficits of object recognition memory. Finally, the procognitive effects were also achieved when dFBr was combined with TC-2403, an α4β2-nAChR agonist.

There are limited data on the efficacy of α4β2-selective ligands for enhancing cognitive flexibility in the ASST. Our previous study demonstrated that TC-2403 facilitated ED set-shifting in rats and that this effect was blocked by DHβE [25]. The improvement in rats’ ED performance was also demonstrated after the administration of 5IA-85380, a β2-nAChR-selective agonist [31]. The current results corroborate these findings by demonstrating the potential of an α4β2-nAChR PAM to facilitate cognitive flexibility in an α4β2-nAChR-dependent manner.

Our study also demonstrated that dFBr was effective in ameliorating delay-induced deficits in object recognition memory and that this activity was also α4β2-dependent. However, the effective dose of dFBr (i.e., 3 mg/kg) was higher than the doses that produced improvement in the ASST (i.e., 0.3 and 1.0 mg/kg). Likewise, TC-2403 facilitated cognitive flexibility at doses of 0.03–0.1 mg/kg [25], while a higher dose of 0.3 mg/kg was necessary to enhance recognition memory (Supplement 1). As a similar trend was previously noted for other nicotinic acting agents (e.g., [27]), it may be suggested that delay-dependent forgetting of object memory is a less sensitive task than the ASST for detecting cognitive enhancement. It should be noted that the results reported here were obtained from male rats only and further studies are required to determine potential sex differences.

In line with our data, the enhancement of recognition memory was observed in rats administered different α4β2-selective agonists. For example, the ability to discriminate between a novel object and a familiar object after a 24-h delay was improved by TC-1734 (AZD3480, ispronicline) in mice [32] and by TC-6683 (AZD1446) in rats [33]. The administration of 0.1 mg/kg TC-2403 reversed 6 h ITI-induced forgetting in rats [29]. Although the efficacy of TC-2403 was also supported in the current study (Supplement 1), the active dose was higher (i.e., 0.3 mg/kg) than that used by McLean et al. [29]. Moreover, 0.3 mg/kg TC-2403 decreased object exploration during the acquisition trial (Supplement 1). On the contrary, dFBr enhanced object recognition in the absence of any deleterious effect on exploratory or locomotor activity, supporting previous results in mice where this PAM (up to 6.0 mg/kg) did not affect open field locomotor activity [21].

The α4β2-PAM not only enhanced the performance of cognitively unimpaired animals but also reversed object recognition deficits in a pharmacological model of schizophrenia based on the administration of ketamine, an NMDA receptor antagonist. The efficacy of α4β2-selective ligands has not been widely assessed in schizophrenia-like animal models. For example, ketamine-induced deficits on a tactile-to-visual cross modal object recognition task and set-shifting task in rats were reversed by the α4β2-nAChR agonists ABT-418 [34] and TC-2403 [25], respectively. Additionally, administration of the β2-nAChR agonist (A-85380) ameliorated object recognition deficits induced by another NMDA receptor antagonist, phencyclidine [35], while NS9283 had favourable effects on phencyclidine-disrupted sensory information [17]. Furthermore, dFBr ameliorated recognition memory deficits induced by scopolamine, a muscarinic receptor antagonist. The full reversal of the deficit was noted at a dose of 1.0 mg/kg dFBr, while a dose of 3.0 mg/kg was necessary to block delay- or ketamine-induced forgetting. According to data in the literature, several α4β2-agonists are also capable of reversing scopolamine-induced memory impairments (e.g., [23, 32, 36]). Likewise, the amnestic effects of scopolamine on the passive avoidance task in rats were reversed by TC-1734 [32], TC-2559 [36], and TC-2403 [23]. The administration of ABT-089 to scopolamine-treated [37] and aged [38] rats reversed spatial learning deficits in the Morris water maze.

Our results also demonstrated that the co-administration of inactive doses of dFBr and TC-2403 can lead to procognitive effects. This finding corroborates previous reports in which dFBr enhanced nicotine-induced antinociception in a mouse model of neuropathic pain [39]. Similarly, NS9283 augmented the antinociceptive properties of the α4β2-agonist ABT-594 in various pain models [40, 41]. Although we are unaware of any data demonstrating that α4β2-PAMs may augment the procognitive activities of selective agonists, this approach was successfully used by combining α7-nAChR PAMs with direct agonists [42, 43]. For example, the co-administration of inactive doses of an α7-nAChR PAM, 3-furan-2-yl-*N*-*p*-tolyl-acrylamide, with orthosteric agonists of α7-nAChR (DMXBA or A-582941) improved object recognition and facilitated attentional set-shifting in rats [42]. Moreover, another α7-nAChR PAM, PNU-120596, enhanced the procognitive efficacy of a subthreshold dose of donepezil, an acetylcholinesterase inhibitor (AChEI) that increases the synaptic concentration of ACh [43]. This strategy may be particularly beneficial in conditions with compromised cholinergic function (e.g., in AD), in which PAMs alone may be ineffective due to scarce ACh levels or when the use of high doses of either agonists or acetylcholinesterase inhibitors may be limited due to adverse side effects.

The potential mechanisms underlying the procognitive effects of α4β2-ligands might be discussed in relation to the known function of α4β2-nAChRs in the regulation of the release of neurotransmitters involved in cognitive processes. For example, α4β2-nAchR activation evoked the release of ACh in the cortex [44]. Moreover, electrochemical recordings of cholinergic transmission revealed that selective stimulation of α4β2-nAChRs evoked transient increases in prefrontal ACh release that may, in turn, predict enhancement of attentional functions [45]. There is also a link between α4β2-induced signalling and glutamate (Glu) release. For example, a selective α4β2-agonist elicited Glu release from hippocampal synaptosomes in a DHβE-sensitive fashion [46]. In line with this finding, in vivo studies demonstrated NS9283-evoked potentiation of nicotine-evoked Glu release in the rat medial PFC [47]. It has also been demonstrated that α4β2-nAchR activation induced dopamine release in the rat PFC, and this effect was blocked by DHβE [48]. It cannot be excluded, however, that other brain regions, e.g., the nucleus accumbens, ventral tegmental area or substantia nigra, may be also implicated in the observed effects of α4β2-nAchR stimulation.

Stimulation of α4β2-nAChRs can also be effective against pathological changes observed in psychiatric disorders, including GABAergic deficits specifically recognized in schizophrenia or Aβ pathology in AD. For example, A–85380, a β2-selective agonist, reversed the epigenetically induced transcriptional downregulation of glutamic acid decarboxylase67 (GAD67) in cortical GABAergic neurons [49]. Interestingly, dFBr prevented the inhibition of α4β2-nAChRs by Aβ1–42 peptides [50]. Thus, the potential disease-modifying properties of α4β2-selective ligands await further studies.

The present study corroborates the concept that α4β2-nAChRs are involved in cognitive processes and demonstrates, for the first time, that α4β2 potentiation improves cognitive flexibility and recognition memory as well as rescues drug-induced cognitive deficits. The strategy based on PAM-induced α4β2 enhancement, either alone or in combination with orthosteric agonists, could offer a useful approach to treat cognitive deficits associated with schizophrenia or AD.

## Electronic supplementary material

Below is the link to the electronic supplementary material.

Supplementary file1 (DOCX 13 kb)

Supplementary file2 (DOCX 19 kb)
