# Supplementary material for: Desformylflustrabromine, a positive allosteric modulator of α4β2-containing nicotinic acetylcholine receptors, enhances cognition in rats
Source: Pharmacol Rep. 2020 Mar 23;72(3):589–99. doi: 10.1007/s43440-020-00092-4 (PMC7329799; doi:10.1007/s43440-020-00092-4)
Supplement: Supplementary file 1 — Supplementary file1 (DOCX 13 kb) [file 43440_2020_92_MOESM1_ESM.docx]

**Supplement 1**

**Fig.S1 The effects of TC-2403 on the novel object recognition task in rats.**

TC-2403 (0, 0.01, 0.1 and 0.3 mg/kg, SC) was administered to rats 30 min prior to the acquisition trial (T1). Data are shown as the mean ± S.E.M. of the discrimination index (DI) during the retention trial. N = 6-8 rats per group. ***p<0.001 significant increase in DI compared to that of the vehicle-treated group.

**Table S1. Object exploration time in the NORT.**

| Treatment (mg/kg) | Acquisition Trial (T1) | | | | | | Retention Trial (T2) | | | | | |
| --- | --- | --- | --- | --- | --- | --- | --- | --- | --- | --- | --- | --- |
|  | Object 1 (s) | | | Object 2 (s) | | | Familiar object (s) | | | Novel object (s) | | |
| Vehicle | 9.7 | + | 0.9 | 9.2 | + | 0.7 | 7.5 | + | 1.6 | 8.5 | + | 1.6 |
| TC-2403 (0.01 mg/kg) | 8.7 | + | 0.6 | 9.4 | + | 0.4 | 7.5 | + | 1.0 | 7.4 | + | 0.6 |
| TC-2403 (0.1 mg/kg) | 7.9 | + | 1.3 | 7.7 | + | 1.3 | 7.8 | + | 0.8 | 12.8 | + | 0.9** |
| TC-2403 (0.3 mg/kg) | 6.9 | + | 0.4 | 6.9 | + | 0.4 | 4.3 | + | 0.6 | 15.4 | + | 1.2*** |

**Results**

There were no significant differences in the time spent exploring two identical objects in the acquisition phase in any group (a two-way ANOVA interaction F[3,26]=0.62, NS, Table 1). However, total object exploration time of rats that were treated with TC-2403 at a dose of 0.3 mg/kg was significantly lower than in the vehicle-treated group (treatment effect: F[3,26]=3,3705, p<0.05, Table 1)

In the retention trial, the vehicle-treated rats spent equal amount of time exploring the novel object and the familiar one (Table 1). This time-induced natural forgetting was ameliorated by the administration of TC-2403 (0.1 and 0.3 mg/kg, two-way ANOVA interaction: F[3,26]=16.79, p<0.001, Table 1). Moreover, the DI for the TC-2403 (0.3 mg/kg)-treated rats was significantly higher than that for the vehicle-treated-treated group (a one-way ANOVA: F[3,26]=11.62, NS, Figure 1).
