# Supplementary material for: Desformylflustrabromine, a positive allosteric modulator of α4β2-containing nicotinic acetylcholine receptors, enhances cognition in rats
Source: Pharmacol Rep. 2020 Mar 23;72(3):589–99. doi: 10.1007/s43440-020-00092-4 (PMC7329799; doi:10.1007/s43440-020-00092-4)
Supplement: Supplementary file 2 — Supplementary file2 (DOCX 19 kb) [file 43440_2020_92_MOESM2_ESM.docx]

**Table 1 S2. Object exploration time in the acquisition (T1) and retention (T2) trials of the novel object recognition task.**

| Treatment (mg/kg) | Acquisition Trial (T1) | | | | | | Retention Trial (T2) | | | | | |
| --- | --- | --- | --- | --- | --- | --- | --- | --- | --- | --- | --- | --- |
|  | Object 1 (s) | | | Object 2 (s) | | | Familiar object (s) | | | Novel object (s) | | |
| vehicle | 4.5 | ± | 0.5 | 4.4 | ± | 0.5 | 4.1 | ± | 0.3 | 4.7 | ± | 0.7 |
| dFBr (1.0) | 4.2 | ± | 0.5 | 4.1 | ± | 0.5 | 3.2 | ± | 0.2 | 3.9 | ± | 0.5 |
| dFBr (3.0) | 4.7 | ± | 0.5 | 4.3 | ± | 0.4 | 4.2 | ± | 0.6 | 10.2 | ± | 0.8*** |
| vehicle + vehicle | 5.7 | ± | 0.8 | 5.6 | ± | 0.9 | 5.3 | ± | 0.7 | 5.6 | ± | 1.0 |
| dFBr (3.0) + vehicle | 5.8 | ± | 0.5 | 6.2 | ± | 0.7 | 3.9 | ± | 1.0 | 11.7 | ± | 0.8*** |
| vehicle + DHβE (3.0) | 5.2 | ± | 0.6 | 5.3 | ± | 0.5 | 4.9 |  | 0.8 | 5.1 |  | 0.8 |
| dFBr (3.0) + DHβE (3.0) | 6.1 | ± | 0.9 | 5.5 | ± | 1.0 | 4.8 | ± | 0.5 | 6.5 | ± | 0.6 |
| vehicle + vehicle | 8.3 | ± | 0.9 | 8.4 | ± | 1.3 | 4.1 | ± | 0.5 | 13.8 | ± | 1.0*** |
| vehicle + ketamine (20) | 7.4 | ± | 1.0 | 7.4 | ± | 0.9 | 8.9 | ± | 0.9 | 7.6 | ± | 0.6 |
| dFBr (1.0) + ketamine (20) | 6.0 | ± | 0.7 | 5.8 | ± | 0.5 | 5.1 | ± | 0.5 | 4.9 | ± | 0.5 |
| dFBr (3.0) + ketamine (20) | 10.3 | ± | 1.0 | 8.9 | ± | 1.0 | 3.7 | ± | 0.6 | 11.4 | ± | 1.6*** |
| vehicle + vehicle | 11.6 | ± | 1.6 | 12.7 | ± | 1.9 | 7.4 | ± | 1.3 | 18.9 | ± | 2.8 |
| vehicle + scop (1.25) | 11.7 | ± | 2.0 | 14.1 | ± | 1.5 | 14.9 | ± | 2.9 | 10.1 | ± | 1.5 |
| dFBr (1.0) + scop (1.25) | 13.8 | ± | 1.1 | 12.6 | ± | 1.5 | 7.7 | ± | 2.1 | 16.6 | ± | 1.2* |
| dFBr (3.0) + scop (1.25) | 9.9 | ± | 2.6 | 11.7 | ± | 2.4 | 9.7 | ± | 2.9 | 20.9 | ± | 2.1** |
| vehicle + vehicle | 9.1 | ± | 0.8 | 9.2 | ± | 0.7 | 7.9 | ± | 1.2 | 8.8 | ± | 1.3 |
| vehice + TC-2403 (0.01) | 8.2 | ± | 0.8 | 8.4 | ± | 0.5 | 7.8 | ± | 0.9 | 8.0 | ± | 0.5 |
| dFBr (1.0) + vehicle | 9.4 | ± | 0.8 | 9.6 | ± | 1.0 | 8.7 | ± | 0.9 | 9.2 | ± | 1.1 |
| dFBr (1.0) + TC-2403 (0.01) | 8.6 | ± | 0.9 | 9.1 | ± | 1.0 | 5.6 | ± | 0.9 | 16.6 | ± | 1.9*** |

Data are presented as the mean ± S.E.M of the exploration time (s). Symbols: *p<0.05, **p<0.01, ***p<0.001: significant difference in time spent exploring the novel object compared with the familiar object. Details are provided in the Figures’ legends and the Materials and Methods section.

**Table 2 S2 Distance travelled in the acquisition (T1) and retention (T2) trials of the novel object recognition task.**

| Treatment (mg/kg) | T1 (m) | | | T2 (m) | | |
| --- | --- | --- | --- | --- | --- | --- |
| vehicle | 11.6 | ± | 1.1 | 11.1 | ± | 0.7 |
| dFBr (1.0) | 9.5 | ± | 1.4 | 8.9 | ± | 0.8 |
| dFBr (3.0) | 9.5 | ± | 0.8 | 10.4 | ± | 1.1 |
| vehicle + vehicle | 10.9 | ± | 1.1 | 10.8 | ± | 0.7 |
| dFBr (3.0) + vehicle | 9.4 | ± | 0.9 | 9.9 | ± | 1.1 |
| vehicle + DHβE (3.0) | 8.9 | ± | 1.2 | 9.6 | ± | 1.0 |
| dFBr (3.0) + DHβE (3.0) | 9.7 | ± | 0.9 | 8.2 | ± | 0.9 |
| vehicle + vehicle | 9.1 | ± | 0.3 | 7.9 | ± | 0.5 |
| vehicle + ketamine (20) | 6.6 | ± | 1.0 | 6.7 | ± | 0.8 |
| dFBr (1.0) + ketamine (20) | 6.4 | ± | 0.9 | 6.7 | ± | 0.5 |
| dFBr (3.0) + ketamine (20) | 5.6 | ± | 1.4 | 5.3 | ± | 0.9 |
| vehicle + vehicle | 15.8 | ± | 1.8 | 15.4 | ± | 1.3 |
| vehicle + scop (1.25) | 12.7 | ± | 1.4 | 14.0 | ± | 1.9 |
| dFBr (1.0) + scop (1.25) | 14.2 | ± | 1.3 | 13.7 | ± | 0.8 |
| dFBr (3.0) + scop (1.25) | 14.9 | ± | 1.5 | 15.3 | ± | 1.2 |
| vehicle + vehicle | 11.9 | ± | 1.0 | 11.4 | ± | 1.4 |
| vehice + TC-2403 (0.01) | 14.7 | ± | 1.0 | 12.9 | ± | 1.2 |
| dFBr (1.0) + vehicle | 15.1 | ± | 1.2 | 14.0 | ± | 1.4 |
| dFBr (1.0) + TC-2403 (0.01) | 13.4 | ± | 0.8 | 13.3 | ± | 1.6 |

Data are presented as the mean ± S.E.M of the travelled distance in meter
